# Rewiring interferon fate: a bidirectional strategy for immune homeostasis

**DOI:** 10.1038/s41392-025-02393-7

**Published:** 2025-09-19

**Authors:** Ben Wielockx, Diego Rodriguez, Peter Mirtschink

**Affiliations:** 1 Institute for Clinical Chemistry and Laboratory Medicine, Faculty of Medicine, Technische Universität Dresden, Dresden, Germany; 2 Experimental Centre, Faculty of Medicine, Technische Universität Dresden, Dresden, Germany

**Keywords:** Innate immune cells, Cell biology

In a recent issue of *Nature*, Witt and colleagues reveal a chromatin-linked immune checkpoint in which SP140 limits interferon responses by repressing RESIST, a protein that stabilizes *Ifnb1* mRNA.^1^ In parallel, SP140 restricts viral replication independently of RESIST, establishing a dual mechanism of antiviral control with broader relevance to immune regulation.

Type I interferons (IFN-I) are central to antiviral defense, orchestrating a transcriptional program that restricts pathogen replication and activates immune effector functions. However, excessive or prolonged IFN-I signaling can disrupt immune homeostasis and contribute to tissue damage, chronic inflammation, or autoimmunity. To balance these opposing outcomes, IFN-I responses are regulated at multiple levels, including transcription, chromatin accessibility, and protein degradation. Recent insights reveal that this multilayered control is essential not only for tailoring responses to pathogens, but also for maintaining tolerance in inflammatory settings. Witt and colleagues revealed an underappreciated layer of IFN-I regulation that operates at the level of mRNA stability rather than transcriptional initiation. Central to this mechanism is the chromatin reader SP140, which was previously identified as a repressor of IFN-I responses although the underlying mechanism remained unclear (as reviewed in^2^). Notably, Witt et al. now demonstrate that SP140 does not directly repress *Ifnb1* transcription. Rather, SP140 silences the *Resist1/2* locus, which encodes RESIST, a novel protein that stabilizes *Ifnb1* transcripts in the cytoplasm.^1^

RESIST operates by engaging the CCR4–NOT deadenylase complex, a central node in mRNA decay. By binding to the CNOT1 and CNOT9 subunits, RESIST disrupts recruitment of the RNA-binding proteins tristetraprolin (TTP) and its paralogs ZFP36L1 and ZFP36L2, which promote rapid decay of AU-rich mRNAs, including *Ifnb1*. Consequently, RESIST prolongs the half-life of *Ifnb1* transcripts, leading to increased IFN-β secretion. Importantly, the genetic deletion of *Resist1/2* in SP140-deficient macrophages restores normal IFN-β levels, while RESIST overexpression increases IFN-I output in mouse and human immune cells. The finding that *Resist1/2* is fully responsible for the sensitivity of *Sp140*^*−/−*^ mice to an IFN-I-dependent *Legionella pneumophila* infection adds an extra dimension to these results. These findings establish RESIST as a novel, post-transcriptional positive regulator of IFN-I, adding a new layer to cytokine regulation.

Additionally, the authors demonstrated that SP140 independently restricts the replication of gamma herpesvirus (MHV68) beyond transcriptional control, revealing an expanded innate defense system involving chromatin-level repression, direct viral restriction, and mRNA surveillance. SP140’s dual role may provide an example of effector-triggered immunity (ETI), a strategy in plant immunity where host factors detect and counteract pathogen effectors.^3^ While this is shown for MHV68 at this point, the authors reveal that SP140 forms nuclear bodies and restricts the virus independently of IFN-I signaling. However, viral antagonism of SP140 unleashes RESIST, which in turn stabilizes *Ifnb1* mRNA and initiates a compensatory IFN-I response. This layered response may allow the host to maintain antiviral defenses, even when SP140 function is impaired. In this context, RESIST could function as a fail-safe amplifier, positioning the SP140-RESIST circuit as a potential mammalian analogue of ETI.

**Fig. 1:**
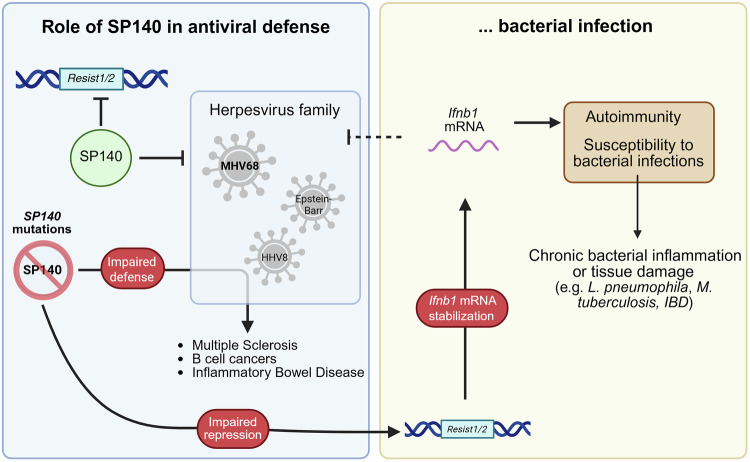
The dual role of SP140 exerts a two-pronged control over type I interferon (IFN-I) responses and pathogen defense. (1) Transcriptional repression: SP140 suppresses the expression of the *Resist1/2* locus, thereby limiting the production of RESIST, a protein that stabilizes *Ifnb1* mRNA in the cytoplasm. By restricting RESIST levels, SP140 prevents excessive stabilization of *Ifnb1* transcripts and curtails prolonged IFN-β secretion, thereby maintaining immune homeostasis. (2) Direct antiviral restriction: Independent of RESIST, SP140 directly inhibits the replication of murine gammaherpesvirus (MHV68), providing an additional layer of innate antiviral defense. Loss of SP140 disrupts both regulatory arms, resulting in increased RESIST expression, enhanced IFN-I activity, and impaired viral restriction. These defects can lead to excessive inflammation and increased susceptibility to viral infections, such as herpesvirus infections, and contribute to the pathogenesis of chronic inflammatory and immune-mediated diseases, including multiple sclerosis, B-cell malignancies, and inflammatory bowel disease (IBD). Furthermore, independent studies have linked SP140 dysfunction to increased susceptibility to bacterial infections and microbiota-driven inflammation, highlighting its broader role in immune regulation and host-microbe balance

From a systems immunology perspective, these findings shift the focus from the transcriptional regulators of IFN-I to the post-transcriptional maintenance of cytokine output. The ability to selectively stabilize *Ifnb1* mRNA without affecting other transcripts, such as *Tnf* or *Il6*, suggests a highly specific mechanism. These insights may help explain why SP140 mutations are associated with immune disorders such as multiple sclerosis, B-cell malignancies, as well as inflammatory bowel disease. Such conditions often feature aberrant IFN-I activity and associations with herpesvirus infections (Fig. 1).

From a clinical perspective, the SP140–RESIST axis could hold therapeutic promise. RESIST activation could boost IFN-I responses in cases of viral evasion or immune suppression. Conversely, reducing RESIST activity or mimicking SP140 function could be beneficial in IFN-driven autoimmune syndromes, where mRNA stabilization contributes to chronic inflammation. However, caution should be taken when approaching these interventions, as excessive IFN-I activity may exacerbate autoimmunity. Conversely, dampening IFN-I signaling could increase the risk of viral reactivation or impaired antiviral defense. Hence, future work should address, whether pharmacological modulation of the SP140–RESIST axis can safely restore immune balance in these settings.

This study raises several important questions. First, RESIST’s apparent specificity in stabilizing *Ifnb1* mRNA but no other well-established TTP targets raises the question of how this selectivity is achieved. It is unclear whether RESIST co-localizes with *Ifnb1* transcripts or the CCR4–NOT complex in a cell-type- or stimulus-specific manner, which could direct stabilization through spatial compartmentalization. It is also conceivable that RESIST may influence the stability of other cytokine or stress-response transcripts under different inflammatory or metabolic conditions. Lastly, identifying viral effectors that antagonize SP140 in vivo, particularly in latent or reactivating herpesvirus infections, could provide insight into host-pathogen coevolution and viral immune evasion strategies.

Answering these questions will clarify how chromatin-level repression interacts with post-transcriptional surveillance mechanisms to determine the strength of innate immune responses. Notably, recent research enhanced our understanding of SP140’s role as a critical regulator in maintaining intestinal immune homeostasis.^4^ SP140-deficient mice develop transmissible dysbiosis, and patients with Crohn’s disease harboring SP140 mutations exhibit blooms of pro-inflammatory *Enterobacteriaceae*. These findings reinforce the importance of SP140 in limiting inappropriate immune activation and preserving the balance between the host and microbiota. The newly defined SP140–RESIST axis may represent an additional post-transcriptional regulatory layer, expanding the model of how SP140 safeguards immune equilibrium through multiple, tiered mechanisms. Witt et al. demonstrated that this axis modulates interferon output in response to *Legionella pneumophila*, not only during viral infection, further suggesting that SP140 operates across pathogen classes (Fig. 1). This aligns with findings by Ji et al., who demonstrated that SP140-deficient mice exhibit elevated IFN-I responses and impaired control of *Listeria monocytogenes* and *Mycobacterium tuberculosis*,^5^ linking chromatin-level interferon repression to antibacterial resistance. Together, the discovery of the SP140–RESIST axis opens new avenues for modulating antiviral and autoimmune responses and highlights the importance of post-transcriptional control in shaping immune homeostasis. Given that genome-wide association studies implicate SP140 in additional immune-related traits, this axis may be relevant in contexts of inflammatory and infectious diseases that have yet to be defined.
